# Conjoined twins in a monochorionic triplet pregnancy after in vitro fertilization: a case report 

**Published:** 2015-11

**Authors:** Marzieh Talebian, Fatemeh Rahimi-Sharbaf, Mahboobeh Shirazi, Batool Teimoori, Narges Izadi-mood, Soheila Sarmadi

**Affiliations:** 1 *Department of Obstetrics and Gynecology, Women Moheb Yas Hospital, Tehran University of Medical Sciences, Tehran, Iran.*; 2 *Department of Obstetrics and Gynecology, Zahedan University of Medical Sciences, Zahedan, Iran.*; 3 *Department of Pathology, Women Moheb Yas Hospital, Tehran University of Medical Sciences, Tehran, Iran.*

**Keywords:** *Conjoined twins*, *Triplet pregnancy*, *Monochorionic-Diamniotic*, *Radiofrequency ablation*

## Abstract

**Background:**

Monozygotic monochorionic triplet pregnancy with conjoined twins is a very rare condition and is associated with many complications.

**Case:**

In this study, we describe a monochorionic–diamniotic triplet pregnancy after in vitro fertilization with an intracytoplasmic sperm injection. At a gestational age of 6 weeks and 4 days of pregnancy one gestational sac was observed, and at a gestational age of 12 weeks and 2 days, triplets with conjoined twins were diagnosed. After consulting with the parents, they chose fetal reduction of the conjoined twins. Selective feticide was successfully performed by radiofrequency ablation at 16 weeks of pregnancy. Unfortunately, the day after the procedure, the membrane ruptured, and 1 week later, all fetuses and placenta were spontaneously aborted.

**Conclusion:**

Monochorionic triplet pregnancy with conjoined twins is very rare. These pregnancies are associated with very serious complications. Intra cytoplasmic sperm injection increases the rate of monozygotic twinning and conjoined twins. Counseling with parents before IVF is very important.

## Introduction

Monozygotic triplet pregnancy is rare, occurring in approximately 4 in 100,000 pregnancies (1). Moreover, conjoined twinning is a rare condition and has been reported at a rate of 1 in 100,000–200,000 live births (2). The presence of conjoined twins in a triplet pregnancy is very rare, occurring in approximately one in a million deliveries (3). In this study, we present an extremely rare condition of a case of conjoined twins in a monochorionic triplet pregnancy.

## Case report

**Figure 1:**
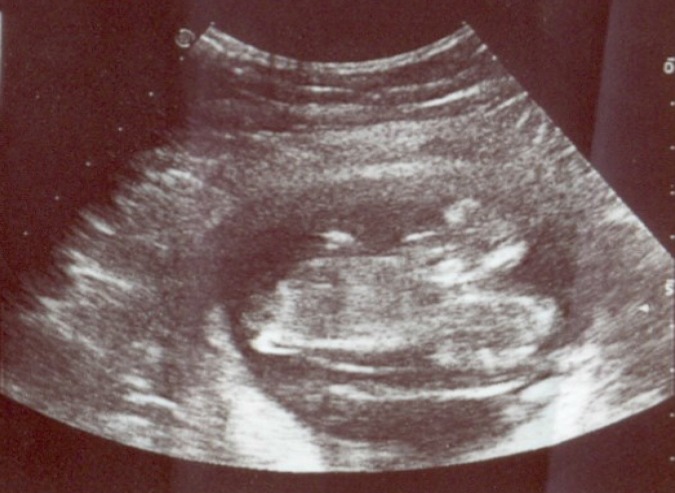
Conjoined fetuses at gestational age of 12 weeks and 3 days.

**Figure 2:**
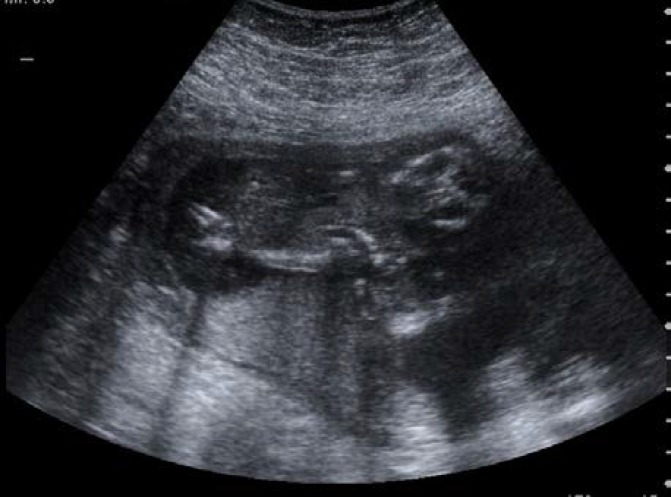
Conjoined fetuses at gestational age of 16 weeks.

**Figure 3:**
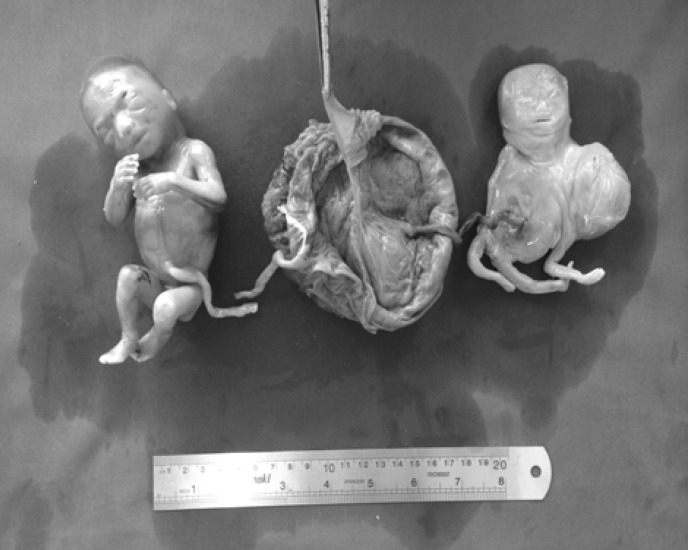
Monochorionic-diamniotic placentation, right normal fetus, left conjoined fetuses with two head and neck, single thorax and abdomen and two upper and two lower limbs

A 38-year-old primigravida woman with conjoined twins in a monochorionic triplet pregnancy was referred to our perinatology center in the women’s Moheb Yas Hospital of the Tehran University of Medical Sciences in May 2014. She had suffered from 22 years of infertility due to male factor. She had a successful pregnancy as a result of an intra cytoplasmic sperm injection cycle (ICSI), and three frozen-thawed blastocysts were transferred into her uterine. Thirty-one days after blastocyst transfer, ultrasound study was performed. One gestational sac with a 6 weeks and 4 days old fetus was detected. After 12 weeks and 2 days of the pregnancy (72 days after blastocyst transfer), screening for aneuploidy was conducted, and in ultrasonography, triplet pregnancy was diagnosed. One amniotic sac contained a single fetus with a nuchal translucency of 1.1mm, while the second amniotic sac contained conjoined fetuses with a gestational age of 12 weeks and 3 days, according to crown rump length (Figure 1). Sixteen days later (88 days after blastocyst transfer: 14 weeks, 4 day), an ultrasound scan was performed, in the ultrasound a single placenta conjoined fetuses with a joined thorax and abdomen, a single heart, and two separated heads were observed. In our center, 98 days after blastocyst transfer (gestational age: 16 weeks), the ultrasound assessment confirmed monochorionic–diamniotic triplet pregnancy with conjoined fetuses with two heads and necks, a single thorax and abdomen, and two upper and lower limbs (Figure 2). The patient and her husband were offered two options: 1) continue the pregnancy or 2) selective feticide of the conjoined fetuses. They chose the second option, and informed consent was obtained from them. Radiofrequency ablation was performed, and the umbilical vein in the intra abdomen of the conjoined twin near the cord insertion was ablated. In a Doppler study of the umbilical cord, the blood flow was observed to stop, and after 35 min, fetal asystole was detected. Five hours later, the patient reported amniotic fluid leakage. Fern test was positive, and in the ultrasound assessment, it was found that the amniotic fluid of the normal fetus had decreased. Premature rupture of the membrane was diagnosed. The patient was observed in the hospital, and 7 days later, spontaneous labor occurred. The fetuses and placenta were aborted. In a gross pathological study, monochorionic–diamniotic placentation was confirmed (Figure 3).

## Discussion

Monochorionic triplet pregnancy with conjoined twins is very rare. These pregnancies are associated with very serious complications. Without intervention, approximately 40% of conjoined twins suffer intrauterine fetal demise. Thirty-five percent of live born-conjoined twins die within the first day (4). 

Parents should be consulted about their options of managing a pregnancy of this nature. Their options are as follows: 1) continuing the pregnancy for future surgery; 2) termination of all pregnancies (this option in our country is illegal, and only malformed fetuses can be terminated); or 3) selective feticide of the conjoined twins. Many parents choose to continue the pregnancies. There are several methods for selective feticide in multiple pregnancies. Intracardiac potassium chloride injection in dichorionic placentation twinning is the most commonly chosen method, but it is impossible in monochorionic placentation because of vascular anastomosis between twins. Selective feticide in monochorionic placentation twinning should be performed by cutting off circulation of the cord in the target fetus. Radiofrequency is a method of feticide in monochorionic-diamniotic pregnancy. 

In this method, cord occlusion is performed by radiofrequency ablation, and it has become the preferred procedure (5-12). In a monochorionic placenta, feticide with potassium chloride may lead to the demise of two fetuses. ICSI increases the rate of monozygotic twinning and conjoined twins (13). However, manipulation of the zona pellucida such as hatching is associated with conjoined twinning (14).

Adverse pregnancy outcome is higher in triplet pregnancy with monochorionic or dichorionic placentation compared with trichorionic pregnancies (15). In triplet pregnancy with conjoined twins, approximately 30% of parents choose to terminate the entire pregnancy and approximately 40% choose selective termination of the conjoined twins (16). By early diagnosis of conjoined twins, better counseling with parents is possible. However, ultrasound examination before 10 weeks of pregnancy is associated with false positives, as a lack of fetal movement may result in the monochorionic twins appearing to be conjoined (17).

By reducing the number of transferred fetuses in IVF, the risk of higher-order multiple pregnancies is not eliminated.

Appropriate counseling with parents before IVF should be conducted, and early ultrasound examination should be recommended. The best time for diagnosis of conjoined twins is the 11–14 weeks of pregnancy (17).

In patients, such as our patient, where surgical separation was not possible, counseling with the parents is very important. However, premature rupture of the membrane and preterm labor is the most serious complication of fetal intervention. 

## Conclusion

Monochorionic triplet pregnancy with conjoined twins is very rare. These pregnancies are associated with very serious complications. Intra cytoplasmic sperm injection increases the rate of monozygotic twinning and conjoined twins. Counseling with parents before IVF is very important. 

## Conflict of interest

We declare that we have no conflict of interest.
